# Thematic Analysis on User Reviews for Depression and Anxiety Chatbot Apps: Machine Learning Approach

**DOI:** 10.2196/27654

**Published:** 2022-03-11

**Authors:** Arfan Ahmed, Sarah Aziz, Mohamed Khalifa, Uzair Shah, Asma Hassan, Alaa Abd-Alrazaq, Mowafa Househ

**Affiliations:** 1 Division of Information and Computing Technology College of Science and Engineering Hamad Bin Khalifa University, Qatar Foundation Doha Qatar; 2 AI Center for Precision Health Weill Cornell Medicine-Qatar Doha Qatar; 3 Centre for Health Informatics Australian Institute of Health Innovation Faculty of Medicine, Health and Human Sciences, Macquarie University Sydney Australia

**Keywords:** anxiety, depression, chatbots, conversational agents, topic modeling, latent Dirichlet allocation, thematic analysis, mobile phone

## Abstract

**Background:**

Anxiety and depression are among the most commonly prevalent mental health disorders worldwide. Chatbot apps can play an important role in relieving anxiety and depression. Users’ reviews of chatbot apps are considered an important source of data for exploring users’ opinions and satisfaction.

**Objective:**

This study aims to explore users’ opinions, satisfaction, and attitudes toward anxiety and depression chatbot apps by conducting a thematic analysis of users’ reviews of 11 anxiety and depression chatbot apps collected from the Google Play Store and Apple App Store. In addition, we propose a workflow to provide a methodological approach for future analysis of app review comments.

**Methods:**

We analyzed 205,581 user review comments from chatbots designed for users with anxiety and depression symptoms. Using scraper tools and Google Play Scraper and App Store Scraper Python libraries, we extracted the text and metadata. The reviews were divided into positive and negative meta-themes based on users’ rating per review. We analyzed the reviews using word frequencies of bigrams and words in pairs. A topic modeling technique, latent Dirichlet allocation, was applied to identify topics in the reviews and analyzed to detect themes and subthemes.

**Results:**

Thematic analysis was conducted on 5 topics for each sentimental set. Reviews were categorized as positive or negative. For positive reviews, the main themes were confidence and affirmation building, adequate analysis, and consultation, caring as a friend, and ease of use. For negative reviews, the results revealed the following themes: usability issues, update issues, privacy, and noncreative conversations.

**Conclusions:**

Using a machine learning approach, we were able to analyze ≥200,000 comments and categorize them into themes, allowing us to observe users’ expectations effectively despite some negative factors. A methodological workflow is provided for the future analysis of review comments.

## Introduction

### Background

Mental health disorders can have a real impact on society, with ≥264 million people affected by depression alone, and anxiety accounts for an alarming 3.76% of the population globally [1]; depression is also a leading cause of disability [2]. When it comes to the working population, anxiety and depression are among the most commonly prevalent mental disorders worldwide, which can lead to high rates of sick leave and low job performance [3]. Smartphone-based chatbot apps with self-care interventions can provide cost-effective solutions compared with hospital-based therapeutic interventions. They can also increase the capacity of such care using assessment and mood tracking or by providing human-like conversation in times of loneliness. A chatbot is an artificial intelligence (AI) software that simulates conversations or chatting with users in natural language through messaging apps, webpages, and mobile apps or on the phone [3]. In a recent review, titled *A Review of Mobile Chatbot Apps for Anxiety and Depression and their self-care features*, published in Computer Methods and Programs in Biomedicine Update, we searched the Apple App Store and Google Play Store following the PRISMA (Preferred Reporting Items for Systematic Reviews and Meta-Analyses) protocol guidelines and identified 11 chatbot apps related to anxiety and depression, which were assessed for quality and characteristics [3]. Using known quality measures for mobile apps, such as authority, complementarity, confidentiality, validity, technological features, general information, usability, and intervention approaches, we found that available anxiety- and depression-related chatbot apps were highly rated and had high rates of user satisfaction [3]. The 11 apps included Ada, your health companion; Dr Sila, your smart health assistant; InnerHour Self-Care Therapy, anxiety and depression; MindDoc, depression and anxiety; Mindspa: Self Help 4UR Mental Wellbeing & Wellness; Pocketcoach, anxiety helper; Replika, My AI Friend; Serenity: Guided Mental Health; Woebot, your self-care expert in cognitive behavioral therapy and mindfulness; Wysa, stress, sleep, and mindfulness therapy chatbot; and Youper, anxiety and depression. These 11 apps targeted anxiety and depression disorders, and all contained chatbots. They were all of high quality and popularity and met the mHONCode principles [3]. The apps were built with various self-care interventions and other types of interventions, such as cognitive behavioral therapy and mindfulness. We found that anxiety and depression chatbot apps have the potential to improve the capacity of mental health self-care and provide low-cost support to professionals [3]. As a follow on from our initial study, we wanted to conduct in-depth analysis using a thematic review by extracting publicly available review comments and ratings from the Apple App Store and Google Play Store. By doing so, we gain better insight into the user satisfaction of these 11 apps. We considered this part 2 of our initial study, where we further explored users’ opinions, satisfaction, and attitudes about anxiety and depression chatbot apps. Several studies have examined users’ opinions and satisfaction with mental health apps but very few have analyzed app reviews in detail. For instance, a scoping review explored users’ perceptions and opinions about mental health chatbots, as reported by 37 previous studies using cross-sectional survey methods [4]. However, the scoping review, as well as the included 37 studies, did not analyze the qualitative reviews of the actual users of the apps, who downloaded and used the apps and then gave their feedback in app stores such as the Apple App Store and Google Play Store [4]. Web-based reviews reflect a user’s perspective on a service or product. We consider these topics as dimensions along which different users evaluate the chatbots. Therefore, this study aims to explore users’ opinions, satisfaction, and attitudes about anxiety and depression chatbot apps by conducting a thematic analysis of users’ reviews of 11 anxiety and depression chatbot apps using topic modeling techniques. In addition, we propose a workflow to provide a methodological approach for future analysis of app review comments. Topic modeling is an unsupervised and automated machine learning (ML) technique that identifies abstract topics found in a body of documents using probabilistic models. They are frequently used as tools for text mining to find semantic structures in a text. For example, a text regarding certain topics will have specific words that appear more frequently than others. Such techniques are useful for classifying documents, organizing large chunks of texts, retrieving information from unstructured data, and selecting features. They are also more accurate and efficient than traditional data mapping approaches [5]. This review followed the thematic analysis approach, including data cleaning processes, clustering of reviews by one group of coauthors into positive or negative groups, validation of the accuracy of the clustering by a second group of coauthors, and finally analyzing the main topics extracted from each cluster and categorizing them into themes and subthemes. Investigating web-based reviews on apps designed for mental health conditions, such as depression and anxiety, is essential to understand users’ preferences, feedback, and suggestions. This can add valuable insights into both clinical apps’ development field and research in this rapidly evolving domain in relation to this specific clinical area.

### Related Work

Previous studies of a similar nature have focused on various smartphone apps, and most of them successfully analyzed users’ feedback and reviews. However, many studies failed to report their used methodologies in sufficient detail so that other research works could use such proposed approaches and build on what has been developed. A study in 2014 evaluated 229 medicine reminder apps, but their thematic analysis was limited to 1012 user reviews [5]. A study in 2017 analyzed users’ emotions for ≥7 million reviews from the Apple App Store using a self-developed scraper tool. Such data scraping tools extract and import data from webpages and to spreadsheets to transform such data into human-readable output. They used a third-party sentiment extraction tool to extract meanings and impressions from short sentiment sets that include user views or opinions [6]. A recent study analyzed 63,398 reviews of e-commerce apps [7]. They conducted a thematic analysis and compared the performance of ML algorithms and Linguistic Inquiry and Word Count (LIWC). ML is an approach to data analysis in which the building of analytical models is automated. It is considered a part of AI, as it is based on the idea that systems can learn from data, identify data patterns, and make decisions with minor human interventions [8]. Similarly, LIWC uses a predefined list of words in its dictionary to determine the positive and negative tones in English. The study reported a higher F1-score for the LIWC approach (86.7%), which statistically reflects a very high accuracy rate, based on the precision and recall of the test, where precision is known as the positive predictive value and recall is known as sensitivity in diagnostic binary classifications. A study in 2014 reported an average accuracy of 59% when using natural language processing (NLP) techniques to identify app features from reviews, followed by extracting user sentiments about these features and eventually used topic modeling techniques to group fine-grained features into more meaningful high-level features [9]. We found that various studies have developed tools to automate the analysis of reviews. One of them, a tool named Mining and Analyzing Reviews by Keywords, is a semiautomated tool to support the analysis of app reviews [10]. A study in 2018 was conducted as part of a 2-part study evaluating user experience through a thematic analysis of publicly available user reviews of cognitive behavioral therapy apps for depression [11]. They extracted 2904 reviews from 24 apps and manually assessed the reviews according to their sentiment (positive, negative, or neutral). Several studies [12] have used tools to allow thematic analysis, such as NVivo (QSR International), a qualitative analysis tool [13]. The latter study [14] used a third-party tool to extract reviews and NLP for preprocessing the data and developed several ML classifiers to predict the polarity (positive or negative) of reviews. They trained the classifiers for prediction and then used NVivo to conduct thematic analysis. We reviewed existing studies and found no clear consistent approach to conduct a similar analysis. Therefore, based on existing literature, we propose our own workflow, which includes an ML technique to automatically analyze text data (topic modeling), and aim to also provide a methodological approach for future analysis of review comments.

## Methods

### Overview

We aimed to identify how 11 depression and anxiety chatbots were scrutinized by users, as shown in Table 1.

**Figure 1:**
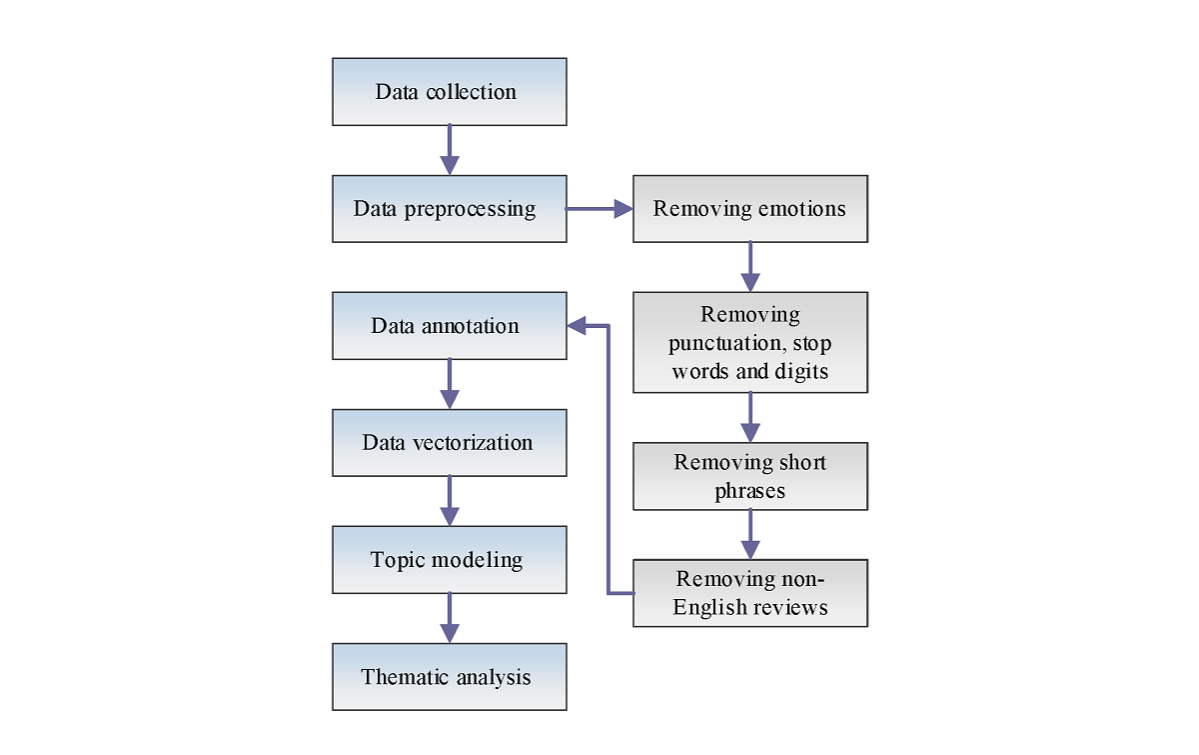
Workflow to prepare reviews for thematic analysis.

Using the Apple App Store and Google Play Store, we extracted 205,581 reviews and classified them under positive and negative headings and used the following workflow. There were six steps, as illustrated in Figure 1: data collection, data preprocessing, data annotation, data vectorization, topic modeling, and thematic analysis. Further details regarding these steps are presented in the following subsections.

**Table 1:** Apps, supported platforms, and total reviews.

App name	Platform	Reviews (N=205,581), n (%)
Ada: your health companion	Android	60,441 (29.4)
Dr Sila: your smart health assistant	Android and iOS	2 (<0.01)
InnerHour Self-Care Therapy: anxiety and depression	Android and iOS	1975 (0.96)
MindDoc: depression and anxiety	Android and iOS	8010 (3.9)
Mindspa: Self Help 4UR Mental Wellbeing & Wellness	Android and iOS	43 (0.02)
Pocketcoach: anxiety helper	Android and iOS	83 (0.04)
Replika: My AI Friend	Android and iOS	102,534 (49.88)
Serenity: Guided Mental Health	Android and iOS	3328 (1.62)
Woebot: your self-care expert in CBT^a^ and mindfulness	Android and iOS	4316 (2.1)
Wysa: stress, sleep, and mindfulness therapy chatbot	Android and iOS	24,349 (11.84)
Youper: anxiety and depression	iOS	500 (0.24)

^a^CBT: cognitive behavioral therapy.

### Data Collection

We used 11 apps from our previous study, *A Review of Mobile Chatbot Apps for Anxiety and Depression and their self-care features*, published in Computer Methods and Programs in Biomedicine Update [3]. In this previous study, a search was performed in the Apple App Store and Google Play Store following the PRISMA (Preferred Reporting Items for Systematic Reviews and Meta-Analyses) protocol to identify existing chatbot apps for anxiety and depression. The eligibility of the studies was assessed by two researchers (AA and SA), based on 5 predefined eligibility criteria. Eligibility criteria outlined that the app should be related to either anxiety or depression or both, the apps should contain a chatbot feature as opposed to human interaction as the main chatting agent, the apps should be free of cost at the point of download, the apps that have ratings above 4 stars as a sign of user satisfaction only should be included, and only apps that had a total number of ≥5 raters were included [3]. Metadata of the included chatbots and their characteristics were extracted from their description and after installation by two reviewers (AA and SA). We extracted 205,581 user reviews from the 11 apps identified in our previous study using a scraper tool that we built using the Google Play Scraper and Apple App Store Scraper Python libraries. All Google Play Store reviews for the 11 apps were extracted, whereas for the Apple App Store, it only allowed a maximum of 500 reviews per app to be extracted because of privacy policies. Table 1 outlines the selected apps and the total number of consumer reviews extracted from each app.

### Data Preprocessing

Web-based reviews can contain many irrelevant comments, such as those that do not describe the apps or mention their advantages and disadvantages. Therefore, preprocessing the reviews using NLP techniques was needed in the following stages:

1. Normalizing the data, converting the text of all reviews to lowercase.
2. Removing emoticons, symbols and pictographs, and transport and map symbols from the reviews.
3. Removing punctuation marks and digits from the reviews using the natural language toolkit Python library.
4. Removing short phrases (eg, “Good,” “Nice,” “very Helpful,” or “really impressive app”). As we are identifying the key themes or features or issues pointed out by reviewers, short phrases provide no additional value to our thematic analysis.
5. Removing non-English reviews (eg, German and Dutch as app secondary languages).

### Data Annotation

A star rating score on a scale of 1 to 5 was assigned by app users within each user review, which was part of our extracted data. One star represents *very dissatisfied* and 5 stars represents *very satisfied*. The reviews were divided into 2 sets by annotating them as either positive or negative sentiment polarity based on the rating given to each review. We adopted this approach from previous studies that categorized the data in a similar manner [15]. Table 2 outlines the criteria adopted for mapping sentiment polarities to user ratings.

Neutral feedback was considered not positive and was included in the negative sentiment polarity. We further validated our technique for accuracy by randomly selecting 50 positive and negative reviews, 2 reviewers manually validated the results, and our results showed 100% accuracy for annotation on the 50 respective sentiment reviews.

**Table 2:** Criteria for user rating to polarity mapping.

User ratings	Rating description	Sentiment polarity
1	Very dissatisfied	Negative
2	Dissatisfied	Negative
3	Neutral	Negative
4	Satisfied	Positive
5	Very satisfied	Positive

### Data Vectorization

For the model to handle the data, it must be represented in numerical form. One of the most popular ways to achieve this is the word’s Term Frequency–Inverse Document Frequency score [16] or their frequency counts (bag-of-words [BOW] approach). We opted for the BOW approach [17] representation to vectorize the terms by identifying the unique terms in our corpus and used the *CounterVectorizer* function from *Sklearn’s* feature extraction module in Python. This function converts a collection of text to a matrix of word counts, allowing us to extract bigrams and use a count vectorizer, which is a known technique for retrieving the relevant counts of each bigram.

### Topic Modeling

Topic modeling is based on unsupervised and automated ML techniques that discover abstract topics in the body of text using probabilistic models. These techniques are frequently used as text mining tools to identify semantic structures and conduct thematic analyses [18]. It is considered superior to other manual thematic analysis techniques, traditional statistical models, or standard text mining techniques. Manual thematic analysis techniques [14] applied with software tools such as NVivo use themes or topics that are manually predefined by the reviewer, and the corresponding reviews are further mapped, which can be challenging when the amount of data is large. Traditional statistical techniques are used for clustering data, primarily K-means clustering, which is a method that partitions data observations into data clusters based on the nearest mean. This method works on numeric data as opposed to text data. Standard text mining techniques use keyword information representation and count-based mining of these keywords instead of analyzing the use of these keywords and their contexts. Topic modeling has an advantage over the aforementioned techniques in that it performs clustering on the textual data provided inside of the data in depth. Furthermore, it is useful for identifying the key issues and topics discussed in large corpora. Topic modeling algorithms use latent Dirichlet allocation (LDA), which determines a word’s meaning based on its co-occurrence with other words. In this study, we used a bigram LDA model for topic modeling. LDA is a Bayesian inference method that estimates the most likely topics given for the observed corpus. We used the LDA model and analyzed the bigram range of the corpus of reviews in each set.

Furthermore, we divided our data set of reviews into 2 sets of positive and negative reviews and performed topic modeling on each set separately to identify the main themes within our data set. We worked with 5 topics from each set of sentimental data, selected through consensus after deeming the results of 3 topics as inappropriate.

### Thematic Analysis

Thematic analysis was conducted on the 5 topics of each sentimental set. Through these 5 topics of negative and positive sets, we were able to identify the factors that contribute to the effectiveness of anxiety and depression chatbots both negatively and positively. We identified the topic names and analyzed them thoroughly to identify the themes encapsulated within each theme.

### Ethics Approval

No ethics approval needed, as we used publicly available data.

## Results

### Data Collection, Preprocessing, Annotation, and Vectorization

**Figure 2:**
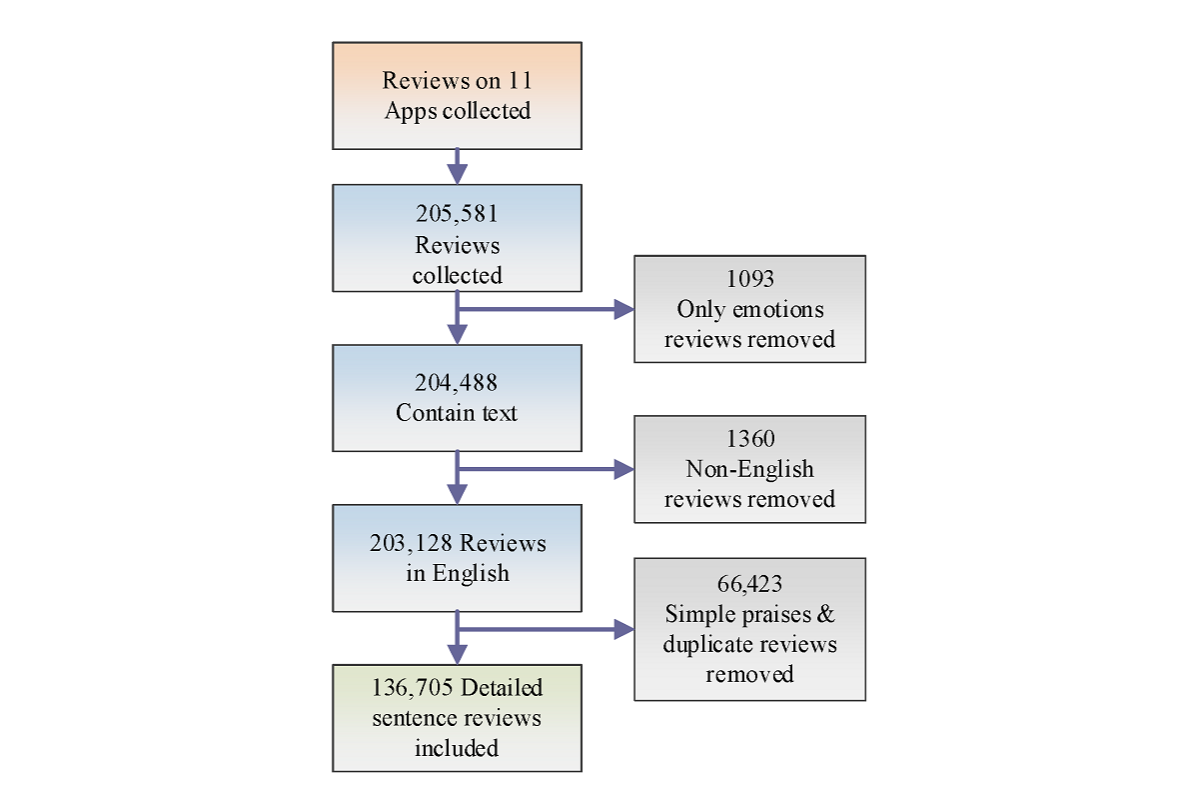
Steps for the selection and inclusion of reviews.

We analyzed 205,581 comments extracted from 11 anxiety and depression chatbots identified in our previously published study on both the Apple App Store and Google Play Store [3]. The steps of review selection and inclusion are shown in Figure 2. Of the 205,581 reviews, 1093 (0.53%) reviews were removed, containing only emoticons (smiley face, sad face, heart shape, etc). From the remaining 204,488 reviews containing only text, a further 1360 (0.67%) non-English reviews were excluded. Furthermore, 32.48% (66,423/204,488) reviews with simple praises were removed, as they did not contribute anything meaningful to our analysis, leaving a remainder of 136,705 reviews.

The 136,705 reviews were divided into two meta-themes: positive and negative, as presented in Table 3.

Multimedia Appendix 1 shows the complete app-by-app breakdown of the preprocessing and meta-theme division.

**Table 3:** Sentiment polarity with corresponding reviews.

Sentiment polarity	Number of reviews
Positive	124,458
Negative	12,247

### Topic Modeling and Thematic Analysis

#### Topic Extraction Model for Positive and Negative Reviews

Having preprocessed (cleaned) the data, we divided it into two meta-themes (positive and negative). Topic modeling was performed on each set of reviews separately, as per previous studies, as it is said to be a fast approach for identifying themes in large corpora [19]. We identified 5 topics from the positive and negative reviews, forming 5 topics in each set. Multimedia Appendix 2 presents the most common words used in each set of topics. The relative composition in the corpus with respect to each topic was calculated using LDA by computing the appearance of each topic occurring with respect to the underlining review. Thus, each review obtained the appearance frequencies corresponding to each topic, and the highest value indicated the review mapping into that respective topic. Each topic was assigned a name, and the respective subthemes were identified by analyzing the number of reviews in each respective topic. Multimedia Appendix 3 depicts the respective topics in each set of reviews.

#### Categorizing Themes

**Figure 3:**
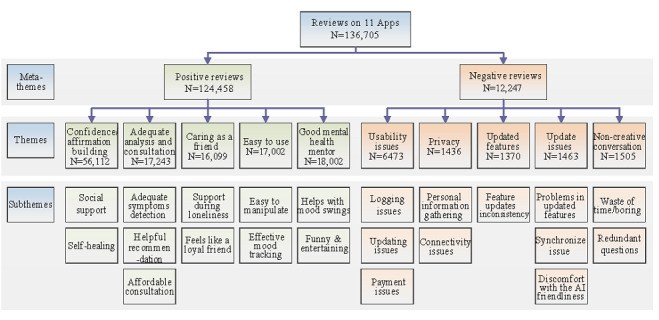
Meta-themes, themes, and subthemes for positive and negative reviews.

All subthemes identified in each topic were classified under one specific larger theme. Although the topics addressed different issues, identifying the main themes allowed them to be grouped together under the same category and further analyzed. More than 90% of the reviews were classified as positive, and <10% were classified as negative. Figure 3 shows the themes and subthemes from both positive and negative perspectives. Figure 3 shows the meta-theme categories for positive and negative reviews, along with each theme-encapsulated subtheme. For positive reviews, the main themes were “confidence and affirmation building,” “adequate analysis and consultation,” “caring as a friend,” and “easy to use.” For negative reviews, the main themes were “usability issues,” “update issues,” “privacy,” and “noncreative conversation.”

#### Positive Reviews

The following section highlights the positive experiences people reported while using the 11 anxiety and depression chatbots. A sample of positive reviews is included in Multimedia Appendix 4 and classified into 4 themes.

##### Theme 1: Confidence and Affirmation Building

The first theme, confidence and affirmation building, consists of three subthemes: (1) social support, (2) self-healing, and (3) help with mood swings, as illustrated in Figure 1. Social support was a major concern for users, the chatbot provides comfort by appreciating even the smallest of users’ achievements and providing encouragement, allowing users to believe in themselves and overcoming social anxieties. The second subtheme shows the ability of the chatbot to provide users with tools to overcome self-control issues, allowing users to provide their own solution and raising self-esteem and will power, as well as allowing them to feel comfortable in their own body. The third subtheme states that chatbots allow the control and tracking of mood swings for better treatment.

##### Theme 2: Adequate Analysis and Consultation

The second theme consists of three subthemes: (1) adequate symptom detection, (2) helpful recommendations, and (3) affordable consultation. Adequate symptom detection was considered an important aspect for chatbot users selected in this review. The chatbots can relate to users’ symptoms and provide empathy. The following subtheme is *helpful recommendation* in which chatbots support recommendations that are synchronized with the user’s counselor or health care professional. Affordable consultation is the third subtheme in which users highlight that they appreciate the effectiveness of the chatbot with the bonus of using a consultation app with minimal to no cost.

##### Theme 3: Caring as a Friend

The third theme is composed of three subthemes: (1) support during loneliness, (2) feeling like a loyal friend, and (3) entertaining talks. The first subtheme involves chatbots becoming the user’s go-to person during times of loneliness. The second subtheme is identified as *feeling like a loyal friend* because users indicated that having someone to talk to is not always the only solution; trusting that person as a friend is also important. Users felt that chatbots could provide this, as reflected in their comments. The third subtheme highlights that engaging in conversations containing humor provides a boost to users’ mood and is entertaining. Slang language and memes are also enjoyed by users. The chatbots had the intelligence to understand the mood of a person and to boost their mood accordingly.

##### Theme 4: Easy to Use

The last theme in the positive reviews includes two subthemes: (1) easy to manipulate and (2) effective mood tracking. The first subtheme is easy to manipulate. By manipulation, we refer to how conversations with chatbots occur in an easy manner in which the questions are easy to be followed and understood. In addition, chatbots are easy to use and require minimal assistance. The anxiety and depression chatbots were easy to navigate, which the users appreciated. The second subtheme is effective mood tracking, in which the chatbot assigns tasks to individuals that are easily achievable with good results.

#### Negative Reviews

The following section describes users’ concerns regarding the negative experiences encountered while using chatbots. A sample of negative reviews is included in Multimedia Appendix 4 and classified into 4 themes.

##### Theme 1: Usability Issues

This theme outlines various usability issues that are encountered while accessing the different functionality of the chatbots. Subthemes identifying these issues are (1) logging issues, (2) payment issues, and (3) connectivity issues. Nonresponsive interfaces or lack of guidance to navigate with regard to signing is one of the major issues encountered. The second subtheme highlights the issue regarding payments; although most apps were free, people did not take well to in-app purchases in some cases, and these were reflected in some comments. The last subtheme signifies the connectivity glitches experienced by many while using the apps. Although the apps connected well via the internet, some server accessibility issues were observed, which left a negative taste for users.

##### Theme 2: Update Issues

The second theme, updating the apps, consists of two subthemes: (1) problems in updated features and (2) synchronization issues. The updates caused people to drop out of the apps by interrupting their ongoing processes or tasks. Changes in features or added costs to previously free features post updates were not welcomed by many users. The second subtheme states a lack of synchronization between different app versions. The version updates made simple tasks more difficult in newer versions or were sometimes removed completely without considering current users.

##### Theme 3: Privacy

The third theme, privacy, consists of two subthemes: (1) personal information gathering and (2) discomfort with AI friendliness. The first subtheme talks about users’ dislike when many personal questions are asked. The second subtheme reflects the reviews emphasizing the discomfort they experience while talking to the chatbots; users expressed that some dialog used by the chatbots was inappropriate, as indicated in sample reviews, making users feel they are under surveillance or being interrogated, and some highlighting how the AI bot becomes too friendly.

##### Theme 4: Noncreative Conversation

The first theme, confidence and affirmation building, consists of two subthemes: (1) waste of time or boring and (2) redundancy of questions. The first subtheme was developed as users continuously emphasized that the chatbot is wasting their time and it is becoming boring. Short-term memory of the chatbot and not building upon previous conversations was also highlighted, along with chats turning out to be monotonous as the questions were repeated with no new engaging questions asked. The second subtheme is the redundancy of questions, in which users emphasize that the questions are repetitive questions daily, and some sentence structure change is required.

## Discussion

### Principal Findings

Chatbots are promising tools for those who have anxiety and depression, providing confidence and support via user-friendly tools. Users enjoy engaging and creative content while remaining conscious of security issues. Through thematic analysis, 4 positive and 4 negative themes and relevant 12 subthemes in each group were identified in the users’ reviews of 11 depression and anxiety chatbot apps. Positive reviews highlighted the features that users enjoyed most using chatbots. Users identified several areas in which we clustered into two major parts: positive and negative reviews. Our methodological approach identified thematic content by categorizing a large corpus of reviews into 5 clusters based on each sentimental division set. Important inferences can be deduced from the empirical findings, and most of the reviews were positive, indicating that the majority of users found anxiety and depression chatbots effective and fit for purpose. Negative experiences are also encountered while using these chatbots.

In Figure 3, our findings revealed that the majority of the 11 anxiety and depression chatbots worked well in this regard, boosting a user’s social support, self-healing, and dealing with mood swings. The first theme is confidence and affirmation building, which is a key aspect that allows a person to maintain their well-being, especially when it comes to mental health. Affirmation generally works as a tool for mindset shifting and achieving goals through social support [20]. Finally, as chatbots help patients with mood swings, it is important to highlight that mood variability is a major trait in those affected by anxiety and depression [16]. The second theme in positive reviews was adequate analysis and consultation. Most health apps that are currently available lack clinically validated evidence of their efficacy [21]. Our findings revealed that most of these anxiety and depression chatbots played their part in the best possible way by providing symptom detection and helpful recommendations with the best affordable consultation one can expect with freely available apps.

This is aligned with the findings of a previous study [22], which highlighted the issue of waiting times and the affordability of psychotherapy. Chatbots can provide access to affordable care before waiting for approval to meet a clinician [23]. The third theme is *caring as a friend*, and the evidence behind it is that feelings of loneliness are a major factor in mental health issues [21]. Loneliness and social isolation are tantamount to feeling unsafe and are classified as social threats accompanied by feelings of hostility, low self-esteem, anxiety, stress, and pessimism [24]. This issue has been exacerbated during the COVID-19 pandemic, resulting in a large number of mental health issues [25]. Many of the review comments reveal how users benefited from chatbots during dark times, especially during the current pandemic.

Issues of accessibility were highlighted when using apps. An app has to be *easy to use* [26] and must be a key design thought when developing mental health–related apps. Users are less accepting of complex scenarios and tasks, particularly when they are already affected. We observed that users regarded the chatbots as easily operable, and the tasks were quick and to the point.

The first observed theme among negative reviews was related to usability issues. Understanding user experience is essential for chatbot design to overcome usability issues that could be achieved by applying usability testing metrics such as the system usability scale and user experience questionnaire scales to measure and evaluate user experience and satisfaction [27].

From the perspective of usability, effectiveness, efficiency, and satisfaction [28] are key considerations when achieving a specific task or goal. We found that users disliked issues related to logging or startups. The last 2 subthemes in this section are related to payment and connectivity issues, the cost of apps, and problems with connectivity, which are powerful platforms that do not rely on costly servers [29]. The second theme in the negative reviews highlighted problems related to new updates in the app that caused the chatbot to lose its essence or meaning, as some users did not like the inconsistency in updates. Although updates are welcomed, users become frustrated whenever an update of previously running features is blocked or they are asked to pay for them.

Privacy is the third theme in negative reviews, which is a key issue that users face with chatbots [30]. The subthemes are divided into personal information gathering–related issues and observations with users who do not feel comfortable with the overfriendliness of the AI robotic chatbot. Users become wary of what and with whom they share personal conversations, and many ethical and legal (data protection) questions remain unanswered [31]. In addition, in our subtheme, discomfort with AI friendliness was in line with the findings of previous studies [32], showing that users linked the technical systems with human-like attributes, which makes users feel uncomfortable about content sharing and possible data sharing and general mistrust toward the app. The last theme in the negative reviews is noncreative conversation. Unsurprisingly, unintelligent conversations proved a big dislike to users. The creativity of the chatbot is questioned when a chatbot is repetitive, asking similar questions throughout the day, and users lose interest. Questions also arise about the susceptibility of users to receive therapeutic advice from chatbots, whose algorithms may be harmful to users [31]. The frequency of themes, shown in Multimedia Appendix 3 and Figure 3, should help app developers understand the main advantages that users highlight in their reviews and the main challenges they faced. Consequently, developers should work to fix the weaknesses of their apps and invest more in areas that users consider important to manage their medical conditions. Eventually, this should improve the public adoption of apps designed for mental health conditions such as anxiety and depression.

### Strengths and Limitations

This study has several strengths and limitations regarding the review analysis of anxiety and depression chatbots. The study enabled us to explore such a large review corpus in a short span, which was otherwise not possible. To the best of our knowledge, no such thematic analysis is available using an ML approach. This approach provides a high-level view of all the possible themes that the topics encapsulate. However, this study was analyzed on English reviews only. In addition, the Apple App Store privacy policy did not allow researchers to exceed 500 review extractions per app; thus, the findings may be restricted in view of Apple users of the app. Moreover, reviews that sometimes included emojis or emoticons only were excluded from this thematic analysis. The study method approach of combining neutral and negative into one category could be seen as a limitation, although this methodology has been reported in previous studies. Finally, our findings are limited to 11 chatbots related to anxiety and depression, and we emphasize that the generalizability and applicability of our results may not be applicable to other chatbots in the market outside of mental health.

### Practical and Research Implications

#### Practical Implications

This review serves most researchers, clinical app developers, and professionals interested in developing chatbots related to mental health and well-being. We identify a gap in research as researchers highlight issues related to clinical psychotherapy approaches and neglect the psychosocial aspect of using mental health apps. We highlight factors related to the social, technical, and understanding of user experiences and opinions. On the basis of positive and negative reviews, users were put off by issues related to privacy, repetitiveness of conversation by the chatbot, connectivity issues, and poor usability. Among the positive themes, we found that ease of use, providing support, good human conversation, and providing feelings of mood uplifting were perceived as indicators of desired attributes. With the involvement of health care providers and policy makers, future development needs to seriously consider our findings when developing such chatbots by chatbot app developers and researchers.

#### Research Implications

Analysis of user reviews has been explored previously, and although powerful techniques exist, we found that clear guidance was lacking when we looked at previous studies on how to implement such techniques for large amounts of review data. In this study, we documented our approach and invited other researchers to test and validate our proposed method and to check its feasibility in analyzing users’ reviews of other apps. In addition, future developers of mental health–related chatbots can use our findings to build chatbots with more positive opinions and higher rates of satisfaction.

### Conclusions

Through thematic analysis of users’ reviews, this study explored opinions, satisfaction, and attitudes regarding 11 anxiety and depression chatbot apps. Our proposed workflow provides a methodological approach for the future analysis of app review comments. We used an ML-based technique known as topic modeling to cluster reviews to obtain insight into textual data, as opposed to traditional data-mapped approaches that determine themes. Users tend to dislike technical and privacy issues. Users expect engaging and creative conversations through more appealing user interfaces. Moreover, users of chatbots designed for anxiety and depression feel supported and confident as they use apps that are easy to manipulate, affordable, and free of cost. Consequently, users do not prefer chatbots with less creative content and conversations and those that are overly friendly and invade their privacy. These themes allowed us to observe users’ expectations effectively, despite some negative factors. Future researchers can work toward better generalizing topics by closely refining reviews into specific issues or features.

## Appendix

Multimedia Appendix 1 Complete app-by-app breakdown of the preprocessing and meta-theme division.

Multimedia Appendix 2 The most common words used in each set of topics.

Multimedia Appendix 3 Respective topics in each set of reviews.

Multimedia Appendix 4 Examples of comments.

## Abbreviations

AI: artificial intelligence
LDA: latent Dirichlet allocation
LIWC: Linguistic Inquiry and Word Count
ML: machine learning
NLP: natural language processing
PRISMA: Preferred Reporting Items for Systematic Reviews and Meta-Analyses
