# Supplementary material for: Thematic Analysis on User Reviews for Depression and Anxiety Chatbot Apps: Machine Learning Approach
Source: JMIR Form Res. 2022 Mar 11;6(3):e27654. doi: 10.2196/27654 (PMC8956988; doi:10.2196/27654)
Supplement: Multimedia Appendix 1 [file formative_v6i3e27654_app1.docx]

# **Appendix A**

Table 4: The 11 Apps and their Total, Positive, and Negative Reviews

| App | Platform | Total Reviews | Total Cleaned Reviews | Positive Reviews | Negative Reviews |
| --- | --- | --- | --- | --- | --- |
| Ada | Android & IOS | 60,441 | 28,215 | 27,407 | 808 |
| Dr Sila | Android | 2 | 1 | 1 |  |
| InnerHours | Android & IOS | 1,975 | 1,489 | 1,309 | 180 |
| MindDoc | Android & IOS | 8,010 | 6,768 | 6,256 | 512 |
| MindSpa | Android & IOS | 43 | 24 | 21 | 3 |
| Pocketcoach | Android & IOS | 83 | 76 | 64 | 12 |
| Replika | Android & IOS | 102,534 | 73,713 | 64,257 | 9,456 |
| Serenity | Android & IOS | 3,328 | 2,607 | 2,524 | 83 |
| Woebot | Android & IOS | 4,316 | 3,788 | 3,582 | 206 |
| Wysa | Android & IOS | 24,349 | 19,564 | 18,606 | 958 |
| Youper | IOS | 500 | 460 | 431 | 29 |
| Total |  | 205,581 | 136,705 | 124,458 | 12,247 |
