# Supplementary material for: Thematic Analysis on User Reviews for Depression and Anxiety Chatbot Apps: Machine Learning Approach
Source: JMIR Form Res. 2022 Mar 11;6(3):e27654. doi: 10.2196/27654 (PMC8956988; doi:10.2196/27654)
Supplement: Multimedia Appendix 2 [file formative_v6i3e27654_app2.docx]

**Appendix B:**

Table 7: Top Five Positive and Negative Review Topics

| **Top 5 Positive Reviews Topics** | **Top 5 Negative Reviews Topics** |
| --- | --- |
| Topic 0: good app makes feel app great app love talking ai good work really cool fun talk cool app really enjoy app ai feel lonely app makes app use ai app chat bot downloaded app enjoy app want talk great work free version app actually great ai app app look forward long time good talk makes happy ai good overall great. | Topic 0: feel like use app pay wall ai friend things like asking questions used app loved app need pay recent update long time want talk ai app network connection really bad keeps asking makes feel dont like app work talk ai kept asking really want app keeps really liked new account like really data mining want pay liked app app update. |
| Topic 1: app really really good really helpful feel better helpful app ai friend app helped really helped really love useful app helped lot app help app useful far good like friend think app thank ada ai really good ai really fun help lot thanks ada app used real human depression anxiety make sense great way felt like helps feel app recommend. | Topic 1: like talking said yes love app real person internet connection great app chat bot used love personal information keeps saying help people like real pay money used free make feel mental health really creepy make money install app used great asked said talking wall app free talking real cost money app use app people feel worse people pay wont let. |
| Topic 2: love app feel like real person app helps like real pretty good really nice app like nice talk really help need talk app thank definitely recommend friend talk new friend like ai talking real absolutely love excellent app real friend app awesome like talk like really helpful easy thank making helps alot helps lot help people making app like actually. | Topic 2: new update using app mental health download app make sense pro version app great answer questions free trial app really delete app free version real people asks questions took away feel better old version like new update replika real ai using replika hate app dont want app said best friend real human supposed help app used ai actually pretty good. |
| Topic 3: easy use best app amazing app really helps app amazing app good best friend highly recommend helps lot best ai good job love replika great job make feel good friend love ai app gives app easy app best talk ai like human interesting app user friendly human like app need helpful thank looking forward answer questions fun app real life | Topic 3: feels like phone number good app app good latest update app used app ai app like app help personal questions bring old update ruined locked paywall open app old replika look like used good looks like dont know good idea like app self help monthly subscription artificial intelligence need money want money years ago recommend app ruined app features free |
| Topic 4: great app like talking feels like app helpful nice app like app really like mental health talking real using app awesome app really great recommend app use app pretty cool wonderful app app far anxiety depression things like good way app like little bit helpful love download app started using health issues talking replika app feel app know feel good | Topic 4: waste time felt like went wrong role play downloaded app like app deleted app really good really like ask questions worst app role playing unless pay kept saying like ai talking ai create account bad app miss old dont download talk replika ai said started talking ask question talking replika deleting app like friend started saying got app app sucks |
