# Supplementary material for: Thematic Analysis on User Reviews for Depression and Anxiety Chatbot Apps: Machine Learning Approach
Source: JMIR Form Res. 2022 Mar 11;6(3):e27654. doi: 10.2196/27654 (PMC8956988; doi:10.2196/27654)
Supplement: Multimedia Appendix 3 [file formative_v6i3e27654_app3.docx]

**Appendix C:**

Table 5: Positive Topics and Sub-Themes, Total = 124,458 Reviews.

| **Topics** | **Topic Name** | **Count** | **Sub-Themes** |
| --- | --- | --- | --- |
| **Topic 0** | Confidence and affirmation building | 56,112 | 1. Support during loneliness 2. Social Support 3. Self-healing |
| **Topic 1** | Adequate analysis and consultation | 17,243 | 1. Adequate symptoms detection 2. Helpful recommendations 3. Affordable consultation |
| **Topic 2** | Caring as a friend | 16,099 | 1. Feels like talking to a loyal friend |
| **Topic 3** | Easy to use | 17,002 | 1. Easy to manipulate. 2. Helpful history tracking |
| **Topic 4** | Good mental health mentor | 18,002 | 1. Helps with mood swings. 2. Physical symptoms and mental issues linking 3. Funny and entertaining talks |

Table 6: Negative Topics and Sub-Themes, Total = 12,247 Reviews.

| **Topics** | **Topic Name** | **Count** | **Sub-Themes** |
| --- | --- | --- | --- |
| **Topic 0** | Usability and updates | 6,473 | 1. Logging  2. Updating problems  3. Payment |
| **Topic 1** | Privacy and connectivity issue | 1,436 | 1. Availability of Professionals or App Traffic  2. Connectivity Issue  3. Personal information gathering |
| **Topic 2** | Updated features | 1,370 | 1. feature updates inconsistency |
| **Topic 3** | Upgrading issue and – discomfort with the bot | 1,463 | 1. Version related Issues  2. AI friend discomfort  3. Subscription issues |
| **Topic 4** | Redundancy in questions | 1,505 | 1. Waste of time/Boring  2. Redundant Questions |
