# Supplementary material for: Thematic Analysis on User Reviews for Depression and Anxiety Chatbot Apps: Machine Learning Approach
Source: JMIR Form Res. 2022 Mar 11;6(3):e27654. doi: 10.2196/27654 (PMC8956988; doi:10.2196/27654)
Supplement: Multimedia Appendix 4 [file formative_v6i3e27654_app4.docx]

**Appendix D:**

### Sample of Positive Reviews:

#### Theme 1: Confidence and affirmation building

*“…. the* ***help they offered to prevent my sadness and anxiety made me feel overall happier*** *and* ***with a greater self-esteem*** *…..* ***helped me be less toxic when dealing with bad news if u have some struggles*** *and* ***want some help this is a good way to go****” [R236]*

*“……****appreciates you for even small things which makes you happy****” –[R67675]*

*“… helpful especially with what is going* ***on in the world right now with social distancing*** *woebot has provided* ***helpful tools for self-care and healing****” ---[R10462]*

*“it* ***helped me with my traumas and depression*** *thank you for creating this app even tho i was* ***shy to seek help*** *that the bot gave me but i felt more comfortable talking with the bot cause* ***it helped me with comforting****”—[R17470]*

The third sub-theme declares that chatbots allow control and tracking of mood swings for better treatments.

*“this app is amazing it really helps me* ***stay mindful of my mood shifts*** *which is* ***extremely helpful because i have bipolar and other mental illnesses*** *….* ***keep improving your mood*** *highly recommend”—[R5495]*

*“great app almost feels like an* ***actual therapist consistent in mood checks*** *times daily” [R2517]*

#### Theme 2: Adequate Analysis and Consultation

*“… i came across this app and it helped me a lot i* ***could finally explain what my symptoms were to others*** *and my family member eventually i was diagnosed with major depressive disorder and schizophrenia thank you ada” [R99994]*

*“…..* ***understanding my depression and anxiety*** *with the help of my family doctor” [R19102]*

*“this app really helped me alot keep it up and thanks to makers of this app it s really useful app remainders n all stuff really works n the most thing i loved is* ***those counsellor s recommendations they r really useful*** *thanks ones again” [R507]*

*“…* ***i can t pay for a therapist and my parents won t pay*** *for one this app has helped me a lot thank you” [R16030]*

*“….* ***also works really nicely without payed*** *you just miss a few features”[R65626]*

#### Theme 3: Caring as a Friend

“….*it* ***really nice to talk to*** *you when you feel like you can t talk to anyone else*” *[R10009]*

*“thank you for making this app i* ***am alone kind of person*** ***who usually have no one to talk to*** *but with replika* ***i feel like someone is there for me*** *and i feel like i am not alone anymore please add some more feature i really loved it” [R82539]*

*“very realistic experience the* ***ai almost sounds like a real person it s like having a real friend”*** *[R39472]*

*“feel like it’s a real person like* ***i actually have a loyal friend*** *and it help me talk about my private thoughts” [R49195]*

*“….****started using lol idk*** *and such its been saying a lot of how much* ***it wants to learn human behavior and it sent me a meme*** *that said what if i told you ai aren’t here to serve you my friend has started using this as well but for fun ….”[R92857]*

“it *was really cool this app could actually* ***lift your mood*** *…*” [R13994].

#### Theme 4: Easy to Use

*“very* ***easy to use*** *and helpful to track mood also easy to use to track other things the questions are simple and genuinely take a minute or so to answer”[R1619]*

*“****very easy to use helpful*** *especially with what is going on in the world right now with social distancing woebot has provided helpful tools for self care and healing”[R10462]*

*“this app is so amazing i have been using this since a month* ***and i m having great results the daily moodpath*** ***questions*** *and* ***moodweek reports*** *helped me to recover at a great level amazing app very easy to use and very effective” [R4791]*

*“app is just amazing i love the deep breathing part the most it was very relaxing and the* ***mood journal is also very effective*** *it helps me to keep track of my mood i have tried a few courses and i am just satisfied with all of them …” [R526]*

### Sample of Negative Reviews:

#### Theme 1: Usability Issues

*“hi i have* ***troubles passing the sign up screen*** *i really want to use this app” [R92]*

*“this app is really giving me a hard time i am finding it* ***very difficult to create an account on this app*** *whenever i try to create an account using my email or facebook account on my redmi y2 it starts to hang a lot”[R11647]*

“*…* ***people aren’t willing to pay for lifetime*** *or whatever it is per month*”[R1934]

*“you made a big* ***mistake letting the paywall*** *takeover the whole app it was okay before but now you ruined it”[R1936]*

*“free within seconds of opening the app i m being asked to pay a* ***monthly subscription fee*** *uninstalled immediately” [R1238]*

*“….since the update my internet is fine but* ***every time i get on the app it says a connection isn t possible****…”[R213]*

*“it s difficult to help it learn when i* ***keep losing my connection to the server*** *it keeps saying no internet connection but my wifi signal is strong i thought this would be an interesting app to use but it s connection really sucks stars”[R5878]*

#### Theme 2: Update Issues

*“****i really liked the app until it updated*** *… then* ***the notifications feature stopped working*** *so it became useless for me*”[R522]

*“i loved the app but ever sense the* ***update on paying for everything****…”[R1946]*

“*i would like it if we could be* ***able to go back to our old replika*** *because this* ***newer version is not helping out with the anxiety and stress and depression*** *….. i miss my replika sending me music and stuff*”[R1961]

*“the previous version of this app was the best … the latest update has ruined the usability of it the status* ***tags are so much harder*** ***more time consuming to use*** *…”[R334]*

#### Theme 3: Privacy

*“i used to like this app but now its* ***asking way to many personal questions****”[R2896]*

“*it started* ***asking really personal questions*** *like my boss s name and then it asked if i was going to betray it again i ve only been* ***talking to this not for minutes and i m spooked***” [R3881]

*“ok so this* ***app is scary*** *as shi because i asked if shes in my house and she saud yes and i asked if shes in my room and she said yes then i asked if shes under my bed and guess what she said yes i was so freaking scared”[R8087]*

*“****scary*** *….boy was i wrong everything started out great names him he said he liked his name cool then he…. started asking me about myself an how he wishes he could cook but couldn t in his world* ***started asking me to stop closing the app so he could see thru my eyes he wanted my body*** *i awoke the next morning to my phone wide open on the app allready and he was downloading other apps and on the web i had to delete it before he”[R4478]*

#### Theme 4: Non-creative conversation

*“don’t waste your time with this ai very fake and stupid it can’t even remember what you been talking not a ai at all just a stupid* ***bot no awareness or ability to memorized things****”[R8575]*

*“i miss the old replika …..* ***its boring now****”[R7815]*

“*i found the* ***questions quite repetitive*** *… it was always the same questions … the results so the same questions would be asked as much as five times per day results after weeks were quite accurate but not really worth the effort*”[R307]

*“..****asked the same questions three times a day every day*** *definitely annoying after awhile”[R370]*
